# Editorial: Mechanobiology and the microenvironment: Computational and experimental approaches

**DOI:** 10.3389/fcell.2022.1054135

**Published:** 2022-10-21

**Authors:** Michael Mak, Aurélie Carlier, Fabian Spill, Andrea Malandrino, Mian Long, Maria Jose Gomez-Benito

**Affiliations:** ^1^ Department of Biomedical Engineering, Yale University, New Haven, CT, United States; ^2^Department of Cell Biology–Inspired Tissue Engineering, MERLN Institute for Technology-Inspired Regenerative Medicine, Maastricht University, Maastricht, Netherlands; ^3^School of Mathematics, University of Birmingham, Birmingham, United Kingdom; ^4^Biomaterials, Biomechanics and Tissue Engineering Group, Department of Materials Science and Engineering, Universitat Politècnica de Catalunya (UPC), Barcelona, Spain; ^5^Institute of Mechanics, Chinese Academy of Sciences, Beijing, China; School of Engineering Science, ^6^University of Chinese Academy of Sciences, Beijing, China; ^7^Mechanical Engineering Departament, University of Zaragoza, Zaragoza, Spain; ^8^Institute of Engineering Research in Aragon, University of Zaragoza, Zaragoza, Aragon, Spain

**Keywords:** microenvironment, extracellular matrix, morphogenesis, bone remodelling and healing, vasculature

The cell microenvironment (ME) varies among a wide range of tissues and influences critical cell processes such as proliferation, differentiation, migration and matrix production. It has been demonstrated that extracellular matrix (ECM) mechanical properties (i.e., stiffness, viscoelasticity (Mierke et al.), adhesiveness and topography) and the mechanical state of the ME influence the outcome of these cellular processes. In recent years, the influence of the ME on cancer progression has been the focus of extensive research. However, how local cell-ME interactions influence tissue level processes, such as vasculogenesis (Edgar et al.; Hermans et al.; Karayaka et al.), tissue morphogenesis (Karayaka et al.) and bone healing and remodelling (Ledoux et al.; Paul et al.), has been understudied. In this research topic collection, exciting new studies shed light on the relationship between the mechanobiology of these processes and the ME, using experimental and computational techniques.

More specifically, the limitations of current measurement techniques make it challenging to directly measure the mechanical stresses of cells and their ME during *in vivo* or *in vitro* experiments, and it is also extremely difficult to relate these stresses to mechanotransduction events, e.g., cells’ ability to sense mechanical stimuli and translate them to biochemical or mechanical signals. The combination of experimental and computational techniques provides a promising and enabling means toward characterizing the cell mechanical ME, as well as to explore different physics-based models to gain a better understanding of the relationship between the ME and cellular events.

The following seven papers in this collection provide new findings and insights to this research topic:

Mierke et al. review the critical role viscoelasticity plays in regulating important cellular processes, such as cell migration and invasion, and discuss whether it can serve as a biomarker. The paper reviews experimental techniques to measure viscoelasticity, and then discusses viscoelasticity on different scales, ranging from the molecular mechanosensory machinery over cellular constituents such as the cytoskeleton to the relation of viscoelasticity with cellular processes such as cell motility or transcriptional changes in the cell. The review finishes by discussing the role of viscoelasticity in multicellular processes and in diseases such as cancer, where viscoelastic properties change in disease progression.

Ledoux et al. provide a unique review on bone remodelling and cytokine dynamics. They discuss relevant mathematical models in this area and link these to the literature presenting clinical data. This review is therefore contributing to closing the gap between mathematical modelling of biomedical problems and clinical practice. Specifically, Ledoux et al. first discuss the criteria for the literature review. They then introduce various bone cell population dynamics models, relevant cytokine pathways, and the parameters for such models based on a systematic review of clinical literature.

Next, Paul et al. show in a combined *in vivo-in silico* study how mechanical stimulation influences the ME in bone defect healing. They focus on bone formation and resorption during fracture healing (reparative and remodelling phases). Through their combined *in vivo* model (mouse model) and computational model (micro-finite element model) they conclude the evolution of bone mineralization is highly influenced by the ME of the bone defect.

Winston et al. analyse how mechanical constraints due to geometrical designs influence tissue remodelling. They build an *in vitro* model with different geometric configurations of stiff standing posts in which mesenchymal tissues remodel. The evolution of the configuration of the tissue is compared to a computational model that incorporates tissue contractility as the main driver of tissue remodelling. They demonstrate the influence of the geometric constraints of the ME in tissue remodelling.

Closely related to these contributions, Hermans et al. report in this research topic that engineered scaffolds with different geometries, recreating MEs with different initial mechanical states, result in different tissue growth and remodelling patterns. The authors identify two different mechanical loading regimes—isotropic and anisotropic—based on *in silico* explorations of the initial scaffold geometry. *In vitro,* bioreactor-maintained cultures of cardiovascular constructs on these scaffold geometries over 14 days evolve differently in terms of structural organisation. They find that the initial scaffold geometry affects enlargement, mechanical outcomes, and collagen and elastin structural evolution. These findings provide evidence for engineering and modulating tissue growth and remodelling *via* control of the initial ME mechanical state.

Edgar et al. present an agent-based model to provide theoretical insight into how endothelial cell force transmission and polarisation at the cellular microscale result in emergent, collective endothelial cell migration and vascular malformations at the tissue macroscale. Interestingly, they show that either high levels of cohesive forces (to maintain a proper endothelial barrier) or mixtures of endothelial cell polarity can create diameter imbalances, persistent flow reversals and functional shunting due to vascular malformations. This study helps demonstrate how blood flow, vascular structure and endothelial cell migration dynamics are linked. The findings also demonstrate the importance of cell-cell adhesion during vascular remodelling, which could be explored as potential targets for the treatment of vascular disease.

Karayaka et al. explore *in vitro* the interplay between mechanical cues and mechanosensitive Notch signalling in regulating the phenotype of vascular smooth muscle cells. They cyclically stretch synthetic and contractile vascular smooth muscle cells, with Notch inhibition and activation. Their results show that cyclic strain decreases Notch signalling and results in a loss of vascular smooth muscle cell contractile features, which could be rescued by activating Notch signalling during cyclic stretching. In addition, their (*in vitro*) data suggest a continuum of vascular smooth muscle phenotypes may exist, ranging from synthetic to contractile, which may also explain current inconsistencies in literature. Taken together, the results highlight the important role of Notch signalling in regulating vascular smooth muscle cell phenotype, putting Notch forward as a potential target in vascular therapy and regenerative medicine.

In summary, a wide range of physiological and pathological phenomena are fundamentally driven by mechanobiological interactions mediated by complex MEs. The ME is inherently complex and critically governed by fundamental physical features, including boundary conditions, rheological properties, anisotropy, and active mechanical processes including flow and contractile forces. Careful consideration of physical cues and interactions and their interplay with biological signalling, enabled by integrated approaches involving computational modelling and mechanical and biophysical experiments, can drive new discoveries and insights in this research topic. New signalling and sensing mechanisms continue to emerge at the intersection of mechanobiology and the microenvironment. Some convergent areas of interest include the roles of viscoelasticity, mechanical conditioning, and memory in driving multiscale cell and tissue organization.

